# Supplementary material for: Design and Evaluation of 223Ra-Labeled and Anti-PSMA Targeted NaA Nanozeolites for Prostate Cancer Therapy—Part II. Toxicity, Pharmacokinetics and Biodistribution
Source: Int J Mol Sci. 2021 May 27;22(11):5702. doi: 10.3390/ijms22115702 (PMC8198605; doi:10.3390/ijms22115702)
Supplement: Supplementary file 1 [file ijms-22-05702-s001.zip › ijms-1216377-supplementary/Supplementary meterials.pdf]

International Journal of *Molecular Sciences*

MDPI

Supplementary Materials

Table S1. Mean fold changes in NF-κB Signaling Target gene expression in LNCaP C4-2 cells after treatment with 50 μg/ml of NaA nenozeolites modified with silane-PEG groups [NaA-silane-PEG] or NaA nanozeolites modified with silane-PEG groups and functionalized with anti-PSMA D2B antibodies [NaA-silane-PEG-D2B] for 24 hours. Mean fold change values from three independent experiments are presented. Fold changes statistically significant in Student's t-test are highlighted in green (up-regulated genes) or red (down-regulated genes). na – not analyzed, fold change and/or P-value cannot be computed due to very low expression level.

Target gene name	Gene	NaA-silane-PEG		NaA-silane-PEG-D2B	
		Mean fold change	P value	Mean fold change	p value
Adrenomedullin	ADM	1,564	0,459	1,579	0,609
Angiotensinogen	AGT	1,419	0,273	1,020	0,846
AKT serine/threonine kinase 1	AKT1	0,908	0,101	1,011	0,874
aldehyde dehydrogenase 3 family member A2	ALDH3A2	1,007	0,915	1,021	0,648
BCL2 Related Protein A1	BCL2A1	-	na	-	na
BCL2 Like 1	BCL2L1	1,134	0,168	1,080	0,401
Baculoviral IAP Repeat Containing 2	BIRC2	1,112	0,429	1,093	0,527
Baculoviral IAP Repeat Containing 3	BIRC3	1,229	0,373	1,076	0,737
Complement **C3**	C3	-	na	-	na
C-C Motif Chemokine Ligand 2	CCL2	0,746	na	-	na
C-C Motif Chemokine Ligand 5	CCL5	0,524	na	0,410	na
C-C Motif Chemokine Ligand 11	CCL11	2,511	0,290	1,795	0,514
C-C Motif Chemokine Ligand 22	CCL22	-	na	-	na
Cyclin D1	CCND1	0,947	0,613	1,076	0,465
C-C chemokine receptor type 5	CCR5	0,504	na	3,742	na
CD40 Molecule	CD40	-	na	-	na
CD69 Molecule	CD69	0,523	na	0,528	na
CD80 Molecule	CD80	1,175	0,769	0,696	0,702
CD83 Molecule	CD83	0,951	0,777	1,013	0,932
cyclin dependent kinase inhibitor 1A	CDKN1A	1,039	0,675	1,126	0,106
complement factor B	CFB	0,990	0,969	1,006	0,960
colony stimulating factor 1	CSF1	1,149	0,762	0,725	0,483
colony stimulating factor 2	CSF2	2,253	0,129	2,347	0,384
colony stimulating factor 3	CSF3	1,205	0,674	0,590	0,479
colony stimulating factor 2 receptor subunit beta	CSF2RB	-	na	-	na
C-X-C Motif Chemokine Ligand 1	CXCL1	-	na	-	na
C-X-C Motif Chemokine Ligand 1	CXCL2	0,337	0,353	0,514	0,443
C-X-C Motif Chemokine Ligand 1	CXCL8	1,342	0,777	1,161	0,885
C-X-C Motif Chemokine Ligand 1	CXCL9	1,001	0,995	0,899	0,607
C-X-C Motif Chemokine Ligand 1	CXCL10	0,881	0,649	1,405	0,237
Epidermal Growth Factor Receptor	EGFR	0,991	0,918	0,919	0,426
Early Growth Response 2	EGR2	4,441	0,074	1,155	0,774
Coagulation Factor III	F3	2,040	0,375	0,690	0,506
Coagulation Factor VIII	F8	1,123	0,349	1,194	0,045
Fas Cell Surface Death Receptor	FAS	1,023	0,836	1,139	0,233
Fas Ligand	FASLG	2,174	0,437	3,595	0,275
Growth Arrest And DNA Damage Inducible Beta	GADD45B	0,905	0,115	0,996	0,868
Intercellular Adhesion Molecule 1	ICAM1	1,295	0,108	1,430	0,050
Interferon Beta 1	IFNB1	0,947	0,909	1,435	na

Interferon gamma	IFNG	-	na	-	na
Interleukin 2	IL2	0,926	na	0,741	na
Interleukin 4	IL4	0,657	0,534	1,239	na
Interleukin 6	IL6	-	na	-	na
Interleukin 15	IL15	0,742	0,579	0,925	0,785
Interleukin 12B	IL12B	0,285	0,385	0,968	0,987
Interleukin 1A	IL1A	0,716	0,303	0,721	0,189
Interleukin 1B	IL1B	1,442	0,522	1,013	0,979
Interleukin 1 Receptor Type 2	IL1R2	0,933	0,739	0,543	0,186
Interleukin 1 Receptor Antagonist	IL1RN	1,008	0,951	**0,572**	**0,013**
Interleukin 2 Receptor Subunit Alpha	IL2RA	-	na	-	na
Insulin	INS	1,061	0,830	0,732	0,416
Interferon Regulatory Factor 1	IRF1	1,154	0,661	1,502	0,259
Lymphotoxin Alpha	LTA	6,144	0,353	1,458	0,514
Lymphotoxin Beta	LTB	0,456	0,270	0,381	0,210
Mitogen-Activated Protein Kinase Kinase 6	MAP2K6	1,088	0,267	1,084	0,195
Matrix Metallopeptidase 9	MMP9	2,375	0,124	2,286	0,140
MYC Proto-Oncogene	MYC	1,103	0,419	1,028	0,690
MYD88 Innate Immune Signal Transduction Adaptor	MYD88	1,169	0,119	**1,146**	**0,048**
Nuclear Receptor Coactivator 3	NCOA3	1,028	0,533	0,908	0,152
Nuclear Factor Kappa B Subunit 1	NFKB1	1,053	0,808	1,180	0,107
Nuclear Factor Kappa B Subunit 2	NFKB2	1,286	0,146	1,227	0,220
NFKB Inhibitor Alpha	NFKBIA	0,888	0,695	1,029	0,918
NAD(P)H Quinone Dehydrogenase 1	NQO1	0,931	0,152	**0,817**	**0,000**
Nuclear receptor subfamily 4, group A, member 2	NR4A2	0,521	0,204	1,480	na
Platelet Derived Growth Factor Subunit B	PDGFB	1,252	0,406	0,814	0,106
Plasminogen activator	PLAU	-	na	-	na
Prostaglandin-endoperoxide synthase 2	PTGS2	-	na	-	na
REL Proto-Oncogene	REL	0,885	0,571	0,930	0,314
RELA proto-oncogene	RELA	1,165	0,171	1,114	0,176
RELB proto-oncogene	RELB	0,986	0,968	0,952	0,904
Selectin E	SELE	2,462	0,128	0,675	0,414
Selectin P	SELP	0,792	na	0,529	na
Synaptosome Associated Protein 25	SNAP25	1,253	na	3,308	na
Superoxide dismutase 2	SOD2	1,072	0,558	1,091	0,269
Signal Transducer And Activator Of Transcription 1	STAT1	1,101	0,373	0,908	0,376
Signal Transducer And Activator Of Transcription 3	STAT3	1,079	0,500	1,137	0,298
signal transducer and activator of transcription 5B	STAT5B	0,953	0,874	1,101	0,687
Tumor necrosis factor	TNF	1,811	na	0,558	na
TNF receptor superfamily member 1B	TNFRSF1B	-	na	-	na
TNF Superfamily Member 10	TNFSF10	1,043	0,538	**0,800**	**0,039**
tumor protein p53	TP53	1,028	0,795	**1,276**	**0,023**
TRAF interacting protein	TRAF2	0,914	0,546	1,104	0,288
Vascular Cell Adhesion Molecule 1	VCAM1	0,525	na	1,201	0,683
X-Linked Inhibitor of Apoptosis	XIAP	1,065	0,613	0,997	0,982

Table S2. Mean fold changes in NF-κB Signaling Target gene expression in DU-145 cells after treatment with 50 μg/ml of NaA nenozeolites modified with silane-PEG groups [NaA-silane-PEG] or NaA nanozeolites modified with silane-PEG groups and functionalized with anti-PSMA D2B antibodies [NaA-silane-PEG-D2B] for 24 hours. Mean fold change values from three independent experiments are presented. Fold changes statistically significant in Student's t-test are highlighted in green (up-regulated genes) or red (down-regulated genes). na – not analyzed, fold change and/or P-value cannot be computed due to very low expression level.

**Target gene name**	**Gene**	**NaA-silane-PEG**		**NaA-silane-PEG-D2B**	
		**Mean fold change**	**P value**	**Mean fold change**	**p value**
Adrenomedullin	ADM	1,146	0,523	1,312	0,209
Angiotensinogen	AGT	0,830	0,061	0,299	0,524
AKT serine/threonine kinase 1	AKT1	1,003	0,950	0,945	0,329
aldehyde dehydrogenase 3 family member A2	ALDH3A2	1,002	0,970	0,963	0,066
BCL2 Related Protein A1	BCL2A1	1,125	0,384	1,249	0,160
BCL2 Like 1	BCL2L1	1,000	0,999	0,980	0,680
Baculoviral IAP Repeat Containing 2	BIRC2	**0,610**	**0,016**	0,784	0,055
Baculoviral IAP Repeat Containing 3	BIRC3	0,848	0,074	0,899	0,157
Complement **C3**	C3	1,270	0,533	0,841	0,667
C-C Motif Chemokine Ligand 2	CCL2	2,726	na	1,764	0,549
C-C Motif Chemokine Ligand 5	CCL5	1,131	0,426	0,906	0,578
C-C Motif Chemokine Ligand 11	CCL11	0,731	0,529	1,337	0,479
C-C Motif Chemokine Ligand 22	CCL22	1,769	0,381	1,164	na
Cyclin D1	CCND1	0,935	0,358	0,983	0,775
C-C chemokine receptor type 5	CCR5	0,237	0,264	0,274	0,301
CD40 Molecule	CD40	1,092	0,602	1,107	0,564
CD69 Molecule	CD69	0,703	0,378	0,494	0,176
CD80 Molecule	CD80	0,311	0,486	0,291	0,479
CD83 Molecule	CD83	1,026	0,781	1,067	0,499
cyclin dependent kinase inhibitor 1A	CDKN1A	**0,607**	**0,000**	**0,673**	**0,001**
complement factor B	CFB	0,978	0,751	0,799	0,057
colony stimulating factor 1	CSF1	**0,847**	**0,016**	**0,749**	**0,001**
colony stimulating factor 2	CSF2	**1,633**	**0,040**	**1,766**	**0,040**
colony stimulating factor 3	CSF3	1,808	0,212	2,351	0,231
colony stimulating factor 2 receptor subunit beta	CSF2RB	-	na	-	na
C-X-C Motif Chemokine Ligand 1	CXCL1	0,852	0,293	1,131	0,391
C-X-C Motif Chemokine Ligand 1	CXCL2	1,000	0,998	1,048	0,477
C-X-C Motif Chemokine Ligand 1	CXCL8	0,974	0,856	1,159	0,357
C-X-C Motif Chemokine Ligand 1	CXCL9	1,239	0,376	1,063	0,882
C-X-C Motif Chemokine Ligand 1	CXCL10	1,427	0,238	**0,446**	**0,023**
Epidermal Growth Factor Receptor	EGFR	1,012	0,538	0,933	0,235
Early Growth Response 2	EGR2	0,733	0,320	0,602	0,176
Coagulation Factor III	F3	1,130	0,180	1,016	0,724
Coagulation Factor VIII	F8	0,949	0,389	0,941	0,095
Fas Cell Surface Death Receptor	FAS	0,889	0,138	0,911	0,143
Fas Ligand	FASLG	0,754	0,618	**0,381**	**0,030**
Growth Arrest And DNA Damage Inducible Beta	GADD45B	0,907	0,109	0,962	0,430
Intercellular Adhesion Molecule 1	ICAM1	1,054	0,080	1,013	0,538
Interferon Beta 1	IFNB1	0,859	0,764	0,813	0,773
Interferon gamma	IFNG	0,194	0,239	0,165	na

Interleukin 2	IL2	-	na	-	na
Interleukin 4	IL4	1,016	0,975	1,543	0,731
Interleukin 6	IL6	1,186	0,178	0,847	0,179
Interleukin 15	IL15	1,028	0,632	0,940	0,197
Interleukin 12B	IL12B	0,528	0,411	0,687	na
Interleukin 1A	IL1A	**0,570**	**0,033**	0,719	0,159
Interleukin 1B	IL1B	**0,495**	**0,014**	0,756	0,140
Interleukin 1 Receptor Type 2	IL1R2	0,651	0,361	0,656	0,311
Interleukin 1 Receptor Antagonist	IL1RN	**1,946**	**0,009**	0,810	0,530
Interleukin 2 Receptor Subunit Alpha	IL2RA	-	na	-	na
Insulin	INS	0,638	0,377	0,883	0,813
Interferon Regulatory Factor 1	IRF1	1,023	0,793	0,967	0,699
Lymphotoxin Alpha	LTA	0,972	0,943	0,337	0,229
Lymphotoxin Beta	LTB	**2,261**	**0,001**	**1,458**	**0,027**
Mitogen-Activated Protein Kinase Kinase 6	MAP2K6	0,933	0,397	**0,857**	**0,007**
Matrix Metallopeptidase 9	MMP9	0,920	0,379	1,030	0,743
MYC Proto-Oncogene	MYC	1,013	0,698	0,982	0,674
MYD88 Innate Immune Signal Transduction Adaptor	MYD88	0,936	0,258	0,935	0,333
Nuclear Receptor Coactivator 3	NCOA3	0,920	0,166	**0,868**	**0,040**
Nuclear Factor Kappa B Subunit 1	NFKB1	1,003	0,870	0,940	0,224
Nuclear Factor Kappa B Subunit 2	NFKB2	**1,053**	**0,044**	1,031	0,409
NFKB Inhibitor Alpha	NFKBIA	0,931	0,146	0,906	0,054
NAD(P)H Quinone Dehydrogenase 1	NQO1	1,000	0,990	**0,940**	**0,005**
Nuclear receptor subfamily 4, group A, member 2	NR4A2	0,992	0,949	1,297	0,604
Platelet Derived Growth Factor Subunit B	PDGFB	1,228	0,099	1,111	0,299
Plasminogen activator	PLAU	**1,104**	**0,010**	1,064	0,182
Prostaglandin-endoperoxide synthase 2	PTGS2	0,986	0,914	0,533	0,121
REL Proto-Oncogene	REL	0,975	0,148	0,921	0,096
RELA proto-oncogene	RELA	0,959	0,309	0,934	0,052
RELB proto-oncogene	RELB	1,226	0,169	1,107	0,463
Selectin E	SELE	0,705	0,517	0,733	0,458
Selectin P	SELP	1,506	na	-	na
Synaptosome Associated Protein 25	SNAP25	1,023	0,841	1,095	0,429
Superoxide dismutase 2	SOD2	1,033	0,337	0,996	0,884
Signal Transducer And Activator Of Transcription 1	STAT1	0,919	0,086	0,890	0,076
Signal Transducer And Activator Of Transcription 3	STAT3	1,053	0,167	**0,910**	**0,048**
signal transducer and activator of transcription 5B	STAT5B	0,986	0,729	1,013	0,816
Tumor necrosis factor	TNF	-	na	1,964	0,406
TNF receptor superfamily member 1B	TNFRSF1B	0,862	0,788	1,004	0,992
TNF Superfamily Member 10	TNFSF10	0,776	0,343	0,669	0,062
tumor protein p53	TP53	1,040	0,670	0,966	0,639
TRAF interacting protein	TRAF2	0,998	0,981	0,949	0,486
Vascular Cell Adhesion Molecule 1	VCAM1	-	na	-	na
X-Linked Inhibitor of Apoptosis	XIAP	**0,847**	**0,022**	0,918	0,094

Table S3. Mean fold changes NF-κB Signaling Target gene expression in RWPE-1 cells after treatment with 50 µg/ml of NaA nenozeolites modified with silane-PEG groups [NaA-silane-PEG] or NaA nanozeolites modified with silane-PEG groups and functionalized with anti-PSMA D2B antibodies [NaA-silane-PEG-D2B] for 24 hours. Mean fold change values from three independent experiments are presented. Fold changes statistically significant in Student's t-test are highlighted in green (up-regulated genes) or red (down-regulated genes). na – not analyzed, fold change and/or P-value cannot be computed due to very low expression level.

**Target gene name**	**Gene**	**NaA-silane-PEG**		**NaA-silane-PEG-D2B**	
		**Mean fold change**	**P value**	**Mean fold change**	**P value**
Adrenomedullin	ADM	1,293	0,557	0,720	0,401
Angiotensinogen	AGT	1,169	na	-	na
AKT serine/threonine kinase 1	AKT1	0,812	0,555	0,688	0,330
aldehyde dehydrogenase 3 family member A2	ALDH3A2	0,988	0,874	1,023	0,685
BCL2 Related Protein A1	BCL2A1	1,647	0,338	0,859	na
BCL2 Like 1	BCL2L1	0,779	0,536	0,669	0,369
Baculoviral IAP Repeat Containing 2	BIRC2	0,928	0,589	0,900	0,379
Baculoviral IAP Repeat Containing 3	BIRC3	1,05	0,733	1,286	0,094
Complement **C3**	C3	0,714	0,426	0,758	0,481
C-C Motif Chemokine Ligand 2	CCL2	1,374	0,474	1,031	0,937
C-C Motif Chemokine Ligand 5	CCL5	1,036	0,803	**0,512**	**0,017**
C-C Motif Chemokine Ligand 11	CCL11	0,539	0,242	0,826	0,818
C-C Motif Chemokine Ligand 22	CCL22	1,03	0,959	0,511	0,271
Cyclin D1	CCND1	0,758	0,322	0,566	0,118
C-C chemokine receptor type 5	CCR5	0,8	na	1,007	0,965
CD40 Molecule	CD40	0,59	0,545	0,411	0,302
CD69 Molecule	CD69	1,13	0,801	0,719	0,644
CD80 Molecule	CD80	0,161	na	0,027	na
CD83 Molecule	CD83	0,955	0,451	**1,326**	**0,013**
cyclin dependent kinase inhibitor 1A	CDKN1A	1,101	0,120	**1,480**	**0,002**
complement factor B	CFB	1,061	0,481	0,994	0,950
colony stimulating factor 1	CSF1	0,949	0,878	0,945	0,864
colony stimulating factor 2	CSF2	0,523	0,377	1,054	0,879
colony stimulating factor 3	CSF3	0,838	0,642	**2,590**	**0,013**
colony stimulating factor 2 receptor subunit beta	CSF2RB	-	na	-	na
C-X-C Motif Chemokine Ligand 1	CXCL1	1,441	0,369	**3,109**	**0,031**
C-X-C Motif Chemokine Ligand 1	CXCL2	1,169	0,328	**1,397**	**0,002**
C-X-C Motif Chemokine Ligand 1	CXCL8	**2,825**	**0,040**	2,873	0,055
C-X-C Motif Chemokine Ligand 1	CXCL9	0,992	0,981	1,127	0,768
C-X-C Motif Chemokine Ligand 1	CXCL10	1,098	0,692	0,771	0,364
Epidermal Growth Factor Receptor	EGFR	0,849	0,554	0,666	0,162
Early Growth Response 2	EGR2	2,439	0,424	1,094	0,955
Coagulation Factor III	F3	0,976	0,927	**0,638**	**0,048**
Coagulation Factor VIII	F8	0,911	0,746	0,825	0,512
Fas Cell Surface Death Receptor	FAS	0,954	0,609	**1,280**	**0,027**
Fas Ligand	FASLG	1,289	0,691	2,017	0,297
Growth Arrest And DNA Damage Inducible Beta	GADD45B	1,107	0,163	**0,765**	**0,009**
Intercellular Adhesion Molecule 1	ICAM1	0,802	0,301	0,808	0,156
Interferon Beta 1	IFNB1	0,603	0,116	0,829	0,775
Interferon gamma	IFNG	-	na	-	na

Interleukin 2	IL2	0,918	na	-	na
Interleukin 4	IL4	1,613	0,137	1,511	0,416
Interleukin 6	IL6	1,252	0,518	1,830	0,082
Interleukin 15	IL15	1,134	0,486	0,971	0,902
Interleukin 12B	IL12B	0,304	0,213	2,131	0,541
Interleukin 1A	IL1A	0,826	0,285	0,966	0,781
Interleukin 1B	IL1B	0,911	0,646	0,954	0,797
Interleukin 1 Receptor Type 2	IL1R2	1,007	0,989	0,827	0,795
Interleukin 1 Receptor Antagonist	IL1RN	1,079	0,475	**0,729**	**0,038**
Interleukin 2 Receptor Subunit Alpha	IL2RA	-	na	-	na
Insulin	INS	0,734	0,412	0,937	0,748
Interferon Regulatory Factor 1	IRF1	0,979	0,961	0,546	0,186
Lymphotoxin Alpha	LTA	0,457	0,408	0,340	0,283
Lymphotoxin Beta	LTB	1,474	0,266	0,679	0,516
Mitogen-Activated Protein Kinase Kinase 6	MAP2K6	0,849	0,253	0,737	0,076
Matrix Metallopeptidase 9	MMP9	1,045	0,850	0,352	0,131
MYC Proto-Oncogene	MYC	1,071	0,685	0,985	0,868
MYD88 Innate Immune Signal Transduction Adaptor	MYD88	1,04	0,285	1,032	0,689
Nuclear Receptor Coactivator 3	NCOA3	0,903	0,589	0,777	0,168
Nuclear Factor Kappa B Subunit 1	NFKB1	0,794	0,533	0,663	0,332
Nuclear Factor Kappa B Subunit 2	NFKB2	0,935	0,767	0,911	0,704
NFKB Inhibitor Alpha	NFKBIA	0,796	0,665	0,613	0,310
NAD(P)H Quinone Dehydrogenase 1	NQO1	1,025	0,599	1,011	0,844
Nuclear receptor subfamily 4, group A, member 2	NR4A2	1,006	0,978	0,877	0,693
Platelet Derived Growth Factor Subunit B	PDGFB	0,707	0,420	0,603	0,176
Plasminogen activator	PLAU	1,008	0,977	0,599	0,148
Prostaglandin-endoperoxide synthase 2	PTGS2	0,904	0,621	0,844	0,366
REL Proto-Oncogene	REL	0,975	0,874	0,737	0,138
RELA proto-oncogene	RELA	0,913	0,725	0,747	0,243
RELB proto-oncogene	RELB	0,986	0,943	1,030	0,916
Selectin E	SELE	0,541	0,233	0,941	0,790
Selectin P	SELP	-	na	-	na
Synaptosome Associated Protein 25	SNAP25	1,787	0,481	1,198	0,824
Superoxide dismutase 2	SOD2	1,051	0,543	**1,221**	**0,046**
Signal Transducer And Activator Of Transcription 1	STAT1	0,867	0,383	0,780	0,182
Signal Transducer And Activator Of Transcription 3	STAT3	0,893	0,478	0,924	0,518
signal transducer and activator of transcription 5B	STAT5B	0,809	0,611	0,683	0,397
Tumor necrosis factor	TNF	-	na	-	na
TNF receptor superfamily member 1B	TNFRSF1B	8,792	0,370	1,480	NaN
TNF Superfamily Member 10	TNFSF10	1,066	0,776	**0,631**	**0,030**
tumor protein p53	TP53	0,718	0,493	0,572	0,224
TRAF interacting protein	TRAF2	0,765	0,513	0,567	0,262
Vascular Cell Adhesion Molecule 1	VCAM1	-	na	-	na
X-Linked Inhibitor of Apoptosis	XIAP	0,818	0,306	0,954	0,766

Table S4. Mean fold changes in NF-κB signaling target gene expression in HPrEC cells after treatment with 50 µg/ml of NaA nenozeolites modified with silane-PEG groups [NaA-silane-PEG] or NaA nanozeolites modified with silane-PEG groups and functionalized with anti-PSMA D2B antibodies [NaA-silane-PEG-D2B] for 24 hours. Mean fold change values from three independent experiments are presented. Fold changes statistically significant in Student's t-test are highlighted in green (up-regulated genes) or red (down-regulated genes). na – not analyzed, fold change and/or P-value cannot be computed due to very low expression level.

**Target gene name**	**Gene**	**NaA-silane-PEG**		**NaA-silane-PEG-D2B**	
		**Mean fold change**	**P value**	**Mean fold change**	**P value**
Adrenomedullin	ADM	1,378	0,058	**1,974**	**0,007**
Angiotensinogen	AGT	1,017	na	0,825	0,838
AKT serine/threonine kinase 1	AKT1	0,907	0,167	**0,636**	**0,003**
aldehyde dehydrogenase 3 family member A2	ALDH3A2	1,202	0,052	**1,615**	**0,000**
BCL2 Related Protein A1	BCL2A1	**1,514**	**0,048**	**0,410**	**0,005**
BCL2 Like 1	BCL2L1	0,822	0,085	**0,688**	**0,012**
Baculoviral IAP Repeat Containing 2	BIRC2	0,935	0,073	**1,102**	**0,024**
Baculoviral IAP Repeat Containing 3	BIRC3	1,102	0,648	**1,703**	**0,001**
Complement **C3**	C3	1,149	0,343	1,058	0,696
C-C Motif Chemokine Ligand 2	CCL2	0,828	na	2,614	na
C-C Motif Chemokine Ligand 5	CCL5	1,523	0,082	1,535	0,071
C-C Motif Chemokine Ligand 11	CCL11	0,617	0,176	0,500	0,360
C-C Motif Chemokine Ligand 22	CCL22	1,967	0,075	0,681	0,501
Cyclin D1	CCND1	0,966	0,236	**0,661**	**0,000**
C-C chemokine receptor type 5	CCR5	1,019	0,967	**3,041**	**0,009**
CD40 Molecule	CD40	0,897	0,535	0,924	0,638
CD69 Molecule	CD69	1,152	0,851	1,798	0,480
CD80 Molecule	CD80	0,335	0,115	1,046	0,950
CD83 Molecule	CD83	1,052	0,546	1,230	0,074
cyclin dependent kinase inhibitor 1A	CDKN1A	**0,898**	**0,011**	**2,222**	**0,000**
complement factor B	CFB	**1,434**	**0,019**	1,267	0,054
colony stimulating factor 1	CSF1	1,006	0,953	1,141	0,134
colony stimulating factor 2	CSF2	0,907	0,830	**0,718**	**0,033**
colony stimulating factor 3	CSF3	1,025	0,950	1,453	0,268
colony stimulating factor 2 receptor subunit beta	CSF2RB	0,909	0,769	0,705	0,589
C-X-C Motif Chemokine Ligand 1	CXCL1	1,091	0,514	**0,398**	**0,000**
C-X-C Motif Chemokine Ligand 1	CXCL2	1,090	0,442	**0,720**	**0,003**
C-X-C Motif Chemokine Ligand 1	CXCL8	1,113	0,677	**0,748**	**0,028**
C-X-C Motif Chemokine Ligand 1	CXCL9	0,902	0,679	0,831	0,487
C-X-C Motif Chemokine Ligand 1	CXCL10	1,035	0,825	0,949	0,385
Epidermal Growth Factor Receptor	EGFR	**0,880**	**0,007**	**1,134**	**0,007**
Early Growth Response 2	EGR2	0,885	0,498	0,573	0,050
Coagulation Factor III	F3	1,053	0,467	**0,815**	**0,022**
Coagulation Factor VIII	F8	0,871	0,486	0,625	0,057
Fas Cell Surface Death Receptor	FAS	0,827	0,079	0,900	0,064
Fas Ligand	FASLG	1,091	0,821	0,493	0,116
Growth Arrest And DNA Damage Inducible Beta	GADD45B	0,886	0,124	**1,484**	**0,014**
Intercellular Adhesion Molecule 1	ICAM1	1,017	0,774	**1,772**	**0,000**
Interferon Beta 1	IFNB1	0,819	0,545	**5,075**	**0,011**
Interferon gamma	IFNG	1,163	0,771	1,325	0,486

Interleukin 2	IL2	1,201	0,698	0,717	0,434
Interleukin 4	IL4	0,277	0,256	0,688	0,350
Interleukin 6	IL6	0,883	0,680	0,875	0,500
Interleukin 15	IL15	0,954	0,719	**2,649**	**0,000**
Interleukin 12B	IL12B	3,400	0,249	3,609	0,250
Interleukin 1A	IL1A	0,851	0,092	0,877	0,089
Interleukin 1B	IL1B	1,091	0,318	**0,652**	0,001
Interleukin 1 Receptor Type 2	IL1R2	1,055	0,496	0,595	0,095
Interleukin 1 Receptor Antagonist	IL1RN	**1,413**	**0,000**	**0,500**	**0,002**
Interleukin 2 Receptor Subunit Alpha	IL2RA	1,829	na	-	na
Insulin	INS	1,782	0,148	0,951	0,874
Interferon Regulatory Factor 1	IRF1	**0,745**	**0,048**	**2,458**	**0,002**
Lymphotoxin Alpha	LTA	1,540	0,079	1,152	0,712
Lymphotoxin Beta	LTB	1,397	0,298	1,075	0,741
Mitogen-Activated Protein Kinase Kinase 6	MAP2K6	0,999	0,991	**4,553**	**0,000**
Matrix Metallopeptidase 9	MMP9	**1,936**	**0,014**	**0,426**	**0,001**
MYC Proto-Oncogene	MYC	0,906	0,077	**0,745**	**0,012**
MYD88 Innate Immune Signal Transduction Adaptor	MYD88	**1,160**	**0,033**	**1,187**	**0,017**
Nuclear Receptor Coactivator 3	NCOA3	**0,904**	**0,009**	**1,472**	**0,004**
Nuclear Factor Kappa B Subunit 1	NFKB1	1,036	0,568	**0,710**	**0,003**
Nuclear Factor Kappa B Subunit 2	NFKB2	1,067	0,494	0,884	0,340
NFKB Inhibitor Alpha	NFKBIA	1,126	0,173	**1,757**	**0,006**
NAD(P)H Quinone Dehydrogenase 1	NQO1	0,984	0,612	**0,770**	**0,018**
Nuclear receptor subfamily 4, group A, member 2	NR4A2	1,174	0,524	1,702	0,114
Platelet Derived Growth Factor Subunit B	PDGFB	0,759	0,116	0,822	0,322
Plasminogen activator	PLAU	0,896	0,420	**0,579**	**0,017**
Prostaglandin-endoperoxide synthase 2	PTGS2	0,911	0,465	**0,501**	**0,000**
REL Proto-Oncogene	REL	1,007	0,902	**1,206**	**0,012**
RELA proto-oncogene	RELA	0,981	0,262	0,912	0,188
RELB proto-oncogene	RELB	1,067	0,392	0,959	0,823
Selectin E	SELE	1,500	0,394	1,019	0,965
Selectin P	SELP	0,611	0,377	1,441	0,654
Synaptosome Associated Protein 25	SNAP25	2,200	0,390	5,586	0,117
Superoxide dismutase 2	SOD2	**1,338**	**0,009**	**1,774**	**0,001**
Signal Transducer And Activator Of Transcription 1	STAT1	**1,142**	**0,009**	**0,862**	**0,018**
Signal Transducer And Activator Of Transcription 3	STAT3	0,967	0,134	**1,115**	**0,007**
signal transducer and activator of transcription 5B	STAT5B	1,039	0,330	0,977	0,530
Tumor necrosis factor	TNF	**1,521**	**0,046**	**1,785**	**0,019**
TNF receptor superfamily member 1B	TNFRSF1B	1,030	0,923	1,807	0,676
TNF Superfamily Member 10	TNFSF10	1,131	0,158	1,191	0,072
tumor protein p53	TP53	0,771	0,085	0,796	0,070
TRAF interacting protein	TRAF2	1,085	0,645	1,025	0,672
Vascular Cell Adhesion Molecule 1	VCAM1	3,039	0,335	0,477	0,144
X-Linked Inhibitor of Apoptosis	XIAP	0,939	0,278	0,998	0,965

Table S5. Mean fold changes in Inflammatory Response & Autoimmunity gene expression in LNCaP C4-2 cells after treatment with 50 µg/ml of NaA nenozeolites modified with silane-PEG groups [NaA-silane-PEG] or NaA nanozeolites modified with silane-PEG groups and functionalized with anti-PSMA D2B antibodies [NaA-silane-PEG-D2B] for 24 hours. Mean fold change values from three independent experiments are presented. Fold changes statistically significant in Student's t-test are highlighted in green (up-regulated genes) or red (down-regulated genes). na – not analyzed, fold change and/or P-value cannot be computed due to very low expression level.

Target gene name	Gene	NaA-silane-PEG		NaA-silane-PEG-D2B	
		Mean fold change	P value	Mean fold change	p value
BCL6 transcription repressor	BCL6	1,012	0,534	0,874	0,020
complement C3	C3	1,070	0,857	1,912	0,185
complement C3a receptor 1	C3AR1	1,215	0,681	1,892	0,131
C-C motif chemokine ligand 2	CCL2	1,457	0,311	1,696	0,126
C-C motif chemokine ligand 3	CCL3	0,881	0,752	1,547	0,316
C-C motif chemokine ligand 4	CCL4	1,286	0,482	2,216	0,045
C-C motif chemokine ligand 5	CCL5	0,929	0,732	1,262	0,156
C-C motif chemokine ligand 7	CCL7	1,068	0,863	1,852	0,125
C-C motif chemokine ligand 8	CCL8	0,961	0,925	1,760	0,184
C-C motif chemokine ligand 11	CCL11	1,104	0,753	1,860	0,051
C-C motif chemokine ligand 13	CCL13	1,392	0,249	1,734	0,050
C-C motif chemokine ligand 16	CCL16	0,988	0,972	1,635	0,092
C-C motif chemokine ligand 17	CCL17	1,129	0,769	1,548	0,232
C-C motif chemokine ligand 19	CCL19	1,219	0,596	1,865	0,143
C-C motif chemokine ligand 21	CCL21	1,086	0,828	1,579	0,236
C-C motif chemokine ligand 22	CCL22	1,096	0,812	1,924	0,051
C-C motif chemokine ligand 23	CCL23	1,030	0,930	1,882	0,087
C-C motif chemokine ligand 24	CCL24	1,225	0,643	2,009	0,163
C-C Motif Chemokine Receptor 1	CCR1	1,124	0,869	2,749	0,196
C-C Motif Chemokine Receptor 2	CCR2	1,118	0,763	1,703	0,110
C-C Motif Chemokine Receptor 3	CCR3	1,089	0,871	2,381	0,099
C-C Motif Chemokine Receptor 4	CCR4	1,042	0,927	1,672	0,214
C-C Motif Chemokine Receptor 7	CCR7	0,872	0,785	1,934	0,127
NADH:ubiquinone oxidoreductase subunit A2	CD14	1,015	0,960	1,589	0,083
CD40 molecule	CD40	1,358	0,491	2,178	0,095
CD40 ligand	CD40LG	1,084	0,865	1,787	0,283
CCAAT Enhancer Binding Protein Beta	CEBPB	1,102	0,341	0,894	0,180
C-reactive protein	CRP	1,141	0,796	2,262	0,128
colony stimulating factor 1	CSF1	0,999	0,993	1,236	0,018
C-X-C Motif Chemokine Ligand 1	CXCL1	1,064	0,872	1,793	0,164
C-X-C Motif Chemokine Ligand 2	CXCL2	0,932	0,894	2,052	0,189
C-X-C Motif Chemokine Ligand 3	CXCL3	1,293	0,467	2,355	0,059
C-X-C Motif Chemokine Ligand 5	CXCL5	0,881	0,514	1,277	0,148
C-X-C Motif Chemokine Ligand 6	CXCL6	1,042	0,934	1,714	0,297
C-X-C Motif Chemokine Ligand 8	CXCL8	1,098	0,677	1,581	0,134
C-X-C Motif Chemokine Ligand 9	CXCL9	0,878	0,807	1,807	0,097
C-X-C Motif Chemokine Ligand 10	CXCL10	1,029	0,950	2,086	0,080
C-X-C motif chemokine receptor 1	CXCR1	1,086	0,863	2,147	0,181
C-X-C motif chemokine receptor 2	CXCR2	0,929	0,800	1,512	0,175
C-X-C motif chemokine receptor 4	CXCR4	0,971	0,844	1,022	0,884

Fas ligand	FASLG	1,147	0,685	2,122	0,085
Fos Proto-Oncogene	FOS	1,066	0,808	1,549	0,061
Interferon Gamma	IFNG	1,112	0,744	1,619	0,208
Interleukin 5	IL5	1,010	0,968	1,547	0,082
Interleukin 6	IL6	0,979	0,956	1,715	0,103
Interleukin 9	IL9	0,826	0,646	1,518	0,168
Interleukin 10	IL10	1,111	0,727	1,622	0,106
Interleukin 15	IL15	1,179	0,617	1,698	0,121
Interleukin 18	IL18	0,943	0,805	1,295	0,322
Interleukin 22	IL22	1,039	0,925	1,909	0,097
interleukin 10 receptor subunit beta	IL10RB	0,983	0,444	1,027	0,584
interleukin 17 receptor A	IL17A	1,035	0,903	1,612	0,166
interleukin 1 alpha	IL1A	0,861	0,308	0,872	0,398
interleukin 1 beta	IL1B	1,012	0,948	1,088	0,571
Interleukin 1 Receptor Type 1	IL1R1	1,137	0,067	0,969	0,136
Interleukin 1 Receptor Accessory Protein	IL1RAP	1,118	0,161	1,018	0,460
Interleukin 1 Receptor Antagonist	IL1RN	0,910	0,671	1,023	0,907
Interleukin 23 Subunit Alpha	IL23A	1,058	0,585	1,180	0,121
Interleukin 23 receptor	IL23R	0,957	0,923	1,800	0,171
Interleukin 6 Receptor	IL6R	**1,115**	**0,014**	1,015	0,473
Integrin Subunit Beta 2	ITGB2	1,024	0,764	1,114	0,126
Kininogen 1	KNG1	1,067	0,869	1,755	0,194
Lymphotoxin Alpha	LTA	1,086	0,770	**1,937**	**0,022**
Lymphotoxin Beta	LTB	1,112	0,792	1,946	0,090
Lymphocyte Antigen 96	LY96	1,242	0,509	1,847	0,083
MYD88 Innate Immune Signal Transduction Adaptor	MYD88	1,011	0,639	**1,100**	**0,000**
Nuclear Factor Kappa B Subunit 1	NFKB1	1,012	0,800	1,016	0,349
Nitric Oxide Synthase 2	NOS2	1,049	0,892	1,532	0,149
Nuclear Receptor Subfamily 3 Group C Member 1	NR3C1	1,306	0,525	1,955	0,100
Prostaglandin-Endoperoxide Synthase 2	PTGS2	1,070	0,857	1,617	0,224
Receptor Interacting Serine/Threonine Kinase 2	RIPK2	**0,935**	**0,004**	0,964	0,279
selectin E	SELE	0,916	0,809	1,700	0,147
TIR domain containing adaptor protein	TIRAP	1,005	0,939	1,022	0,240
toll like receptor 1	TLR1	0,966	0,935	1,853	0,243
toll like receptor 2	TLR2	0,852	0,747	1,825	0,226
toll like receptor 3	TLR3	1,321	0,391	1,577	0,229
toll like receptor 4	TLR4	0,821	0,644	1,689	0,193
toll like receptor 5	TLR5	0,965	0,897	1,358	0,224
toll like receptor 6	TLR6	0,891	0,693	1,263	0,371
toll like receptor 7	TLR7	1,125	0,769	1,996	0,120
toll like receptor 9	TLR9	1,113	0,706	1,675	0,101
tumor necrosis factor	TNF	1,024	0,934	1,419	0,153
TNF Superfamily Member 14	TNFSF14	1,229	0,620	1,864	0,136
Toll Interacting Protein	TOLLIP	1,032	0,330	0,972	0,086

Table S6. Mean fold changes in Inflammatory Response & Autoimmunity gene expression in DU-145 cells after treatment with 50 µg/ml of NaA nenozeolites modified with silane-PEG groups [NaA-silane-PEG] or NaA nanozeolites modified with silane-PEG groups and functionalized with anti-PSMA D2B antibodies [NaA-silane-PEG-D2B] for 24 hours. Mean fold change values from three independent experiments are presented. Fold changes statistically significant in Student's t-test are highlighted in green (up-regulated genes) or red (down-regulated genes). na – not analyzed, fold change and/or P-value cannot be computed due to very low expression level.

Target gene name	Gene	NaA-silane-PEG		NaA-silane-PEG-D2B	
		Mean fold change	P value	Mean fold change	p value
BCL6 transcription repressor	BCL6	1,050	0,477	0,909	0,177
complement C3	C3	1,345	0,349	0,712	0,284
complement C3a receptor 1	C3AR1	1,206	0,740	1,374	0,544
C-C motif chemokine ligand 2	CCL2	1,426	0,450	0,777	0,659
C-C motif chemokine ligand 3	CCL3	1,463	0,217	1,461	0,277
C-C motif chemokine ligand 4	CCL4	0,657	na	0,542	0,065
C-C motif chemokine ligand 5	CCL5	1,391	0,402	1,280	0,511
C-C motif chemokine ligand 7	CCL7	1,115	0,740	0,859	0,648
C-C motif chemokine ligand 8	CCL8	0,940	0,947	0,471	na
C-C motif chemokine ligand 11	CCL11	0,318	0,274	0,488	0,487
C-C motif chemokine ligand 13	CCL13	0,610	0,477	0,630	0,467
C-C motif chemokine ligand 16	CCL16	-	na	-	na
C-C motif chemokine ligand 17	CCL17	-	na	0,129	na
C-C motif chemokine ligand 19	CCL19	**0,331**	**0,021**	0,504	0,296
C-C motif chemokine ligand 21	CCL21	1,050	0,879	0,675	na
C-C motif chemokine ligand 22	CCL22	0,920	0,706	1,018	na
C-C motif chemokine ligand 23	CCL23	0,969	0,966	3,234	0,337
C-C motif chemokine ligand 24	CCL24	1,555			na
C-C Motif Chemokine Receptor 1	CCR1	1,280	0,800	2,156	0,463
C-C Motif Chemokine Receptor 2	CCR2	1,451	0,528	1,975	0,395
C-C Motif Chemokine Receptor 3	CCR3	1,359	0,616	3,179	0,350
C-C Motif Chemokine Receptor 4	CCR4	0,236	0,344	1,108	0,952
C-C Motif Chemokine Receptor 7	CCR7	6,940	0,347	3,621	0,462
NADH:ubiquinone oxidoreductase subunit A2	CD14	0,916	0,762	1,074	0,681
CD40 molecule	CD40	1,180	0,370	1,068	0,705
CD40 ligand	CD40LG	0,598	0,140	0,419	0,058
CCAAT Enhancer Binding Protein Beta	CEBPB	0,909	0,428	0,929	0,354
C-reactive protein	CRP	-	na	-	na
colony stimulating factor 1	CSF1	0,858	0,067	**0,793**	**0,023**
C-X-C Motif Chemokine Ligand 1	CXCL1	0,852	0,253	1,084	0,513
C-X-C Motif Chemokine Ligand 2	CXCL2	1,035	0,578	1,058	0,445
C-X-C Motif Chemokine Ligand 3	CXCL3	1,021	0,804	1,017	0,845
C-X-C Motif Chemokine Ligand 5	CXCL5	1,074	0,608	1,138	0,390
C-X-C Motif Chemokine Ligand 6	CXCL6	0,969	0,648	0,936	0,416
C-X-C Motif Chemokine Ligand 8	CXCL8	1,006	0,956	1,168	0,271
C-X-C Motif Chemokine Ligand 9	CXCL9	0,883	0,827	1,507	0,230
C-X-C Motif Chemokine Ligand 10	CXCL10	0,983	0,979	0,610	0,390
C-X-C motif chemokine receptor 1	CXCR1	-	na	-	na
C-X-C motif chemokine receptor 2	CXCR2	0,839	0,452	0,958	0,750
C-X-C motif chemokine receptor 4	CXCR4	1,197	0,223	1,023	0,788

Fas ligand	FASLG	0,308	0,493	0,761	0,894
Fos Proto-Oncogene	FOS	1,092	0,429	1,057	0,594
Interferon Gamma	IFNG	0,622	na	-	na
Interleukin 5	IL5	0,565	0,291	0,428	0,254
Interleukin 6	IL6	1,244	0,151	0,883	0,344
Interleukin 9	IL9	0,195	0,076	0,474	0,220
Interleukin 10	IL10	0,787	0,519	2,477	na
Interleukin 15	IL15	1,135	0,194	0,985	0,848
Interleukin 18	IL18	0,910	0,177	0,899	0,137
Interleukin 22	IL22	1,363	0,433	1,130	0,641
interleukin 10 receptor subunit beta	IL10RB	1,059	0,587	0,936	0,520
interleukin 17 receptor A	IL17A	-	na	-	na
interleukin 1 alpha	IL1A	0,735	0,240	0,753	0,270
interleukin 1 beta	IL1B	0,544	0,131	0,520	0,128
Interleukin 1 Receptor Type 1	IL1R1	0,977	0,660	**0,867**	**0,042**
Interleukin 1 Receptor Accessory Protein	IL1RAP	0,987	0,679	0,996	0,943
Interleukin 1 Receptor Antagonist	IL1RN	1,369	0,273	0,866	0,363
Interleukin 23 Subunit Alpha	IL23A	1,089	0,530	1,010	0,937
Interleukin 23 receptor	IL23R	-	na	-	na
Interleukin 6 Receptor	IL6R	1,139	0,240	0,890	0,288
Integrin Subunit Beta 2	ITGB2	1,112	0,276	1,021	0,813
Kininogen 1	KNG1	1,987	na	2,329	na
Lymphotoxin Alpha	LTA	0,773	0,757	0,735	0,774
Lymphotoxin Beta	LTB	**2,037**	**0,009**	**1,358**	**0,023**
Lymphocyte Antigen 96	LY96	1,023	0,891	1,181	0,307
MYD88 Innate Immune Signal Transduction Adaptor	MYD88	0,950	0,451	0,951	0,347
Nuclear Factor Kappa B Subunit 1	NFKB1	0,958	0,367	0,919	0,165
Nitric Oxide Synthase 2	NOS2	0,955	0,831	0,671	0,253
Nuclear Receptor Subfamily 3 Group C Member 1	NR3C1	0,931	0,241	**0,884**	**0,009**
Prostaglandin-Endoperoxide Synthase 2	PTGS2	1,038	0,893	0,888	0,691
Receptor Interacting Serine/Threonine Kinase 2	RIPK2	0,990	0,826	0,995	0,918
selectin E	SELE	1,216	0,717	0,680	0,587
TIR domain containing adaptor protein	TIRAP	0,817	0,098	0,971	0,777
toll like receptor 1	TLR1	**0,830**	**0,044**	**0,719**	**0,003**
toll like receptor 2	TLR2	0,981	0,918	1,094	0,506
toll like receptor 3	TLR3	1,017	0,900	1,072	0,439
toll like receptor 4	TLR4	0,853	0,750	3,359	0,067
toll like receptor 5	TLR5	0,826	0,382	0,686	0,073
toll like receptor 6	TLR6	0,927	0,171	0,964	0,470
toll like receptor 7	TLR7	0,514	0,141	0,984	0,970
toll like receptor 9	TLR9	0,565	na	1,693	na
tumor necrosis factor	TNF	1,322	0,455	4,934	na
TNF Superfamily Member 14	TNFSF14	1,137	0,789	1,076	0,876
Toll Interacting Protein	TOLLIP	0,993	0,950	0,931	0,339

Table S7. Mean fold changes in Inflammatory Response & Autoimmunity gene expression in RWPE-1 cells after treatment with 50 µg/ml of NaA nenozeolites modified with silane-PEG groups [NaA-silane-PEG] or NaA nanozeolites modified with silane-PEG groups and functionalized with anti-PSMA D2B antibodies [NaA-silane-PEG-D2B] for 24 hours. Mean fold change values from three independent experiments are presented. Fold changes statistically significant in Student's t-test are highlighted in green (up-regulated genes) or red (down-regulated genes). na – not analyzed, fold change and/or P-value cannot be computed due to very low expression level.

**Target gene name**	**Gene**	**NaA-silane-PEG**		**NaA-silane-PEG-D2B**	
		**Mean fold change**	**P value**	**Mean fold change**	**p value**
BCL6 transcription repressor	BCL6	1,112	0,484	1,127	0,205
complement C3	C3	0,498	0,116	0,911	0,551
complement C3a receptor 1	C3AR1	-	na	1,037	0,971
C-C motif chemokine ligand 2	CCL2	1,099	0,854	1,109	0,817
C-C motif chemokine ligand 3	CCL3	1,326	0,731	1,652	0,562
C-C motif chemokine ligand 4	CCL4	1,062	na	0,953	**na**
C-C motif chemokine ligand 5	CCL5	0,918	0,543	**0,410**	**0,001**
C-C motif chemokine ligand 7	CCL7	1,517	0,178	1,292	0,703
C-C motif chemokine ligand 8	CCL8	-	na	-	na
C-C motif chemokine ligand 11	CCL11	0,846	0,675	2,925	0,050
C-C motif chemokine ligand 13	CCL13	8,211	0,456	1,367	0,823
C-C motif chemokine ligand 16	CCL16	0,632	na	-	na
C-C motif chemokine ligand 17	CCL17	1,909	0,390	1,614	0,376
C-C motif chemokine ligand 19	CCL19	-	na	-	na
C-C motif chemokine ligand 21	CCL21	0,880	0,915	-	na
C-C motif chemokine ligand 22	CCL22	2,415	0,081	1,144	0,643
C-C motif chemokine ligand 23	CCL23	1,681	na	1,512	0,267
C-C motif chemokine ligand 24	CCL24	0,719	0,141	**1,565**	**0,028**
C-C Motif Chemokine Receptor 1	CCR1	3,468	0,225	1,817	0,640
C-C Motif Chemokine Receptor 2	CCR2	2,748	0,458	5,004	0,259
C-C Motif Chemokine Receptor 3	CCR3	0,826	0,914	1,055	0,980
C-C Motif Chemokine Receptor 4	CCR4	1,465	0,672	0,784	0,816
C-C Motif Chemokine Receptor 7	CCR7	0,730	0,556	0,918	0,726
NADH:ubiquinone oxidoreductase subunit A2	CD14	0,945	0,802	0,883	0,619
CD40 molecule	CD40	0,902	0,426	0,796	0,147
CD40 ligand	CD40LG	0,807	0,581	0,609	0,495
CCAAT Enhancer Binding Protein Beta	CEBPB	1,191	0,313	1,247	0,259
C-reactive protein	CRP	-	na	-	na
colony stimulating factor 1	CSF1	0,857	0,053	0,879	0,495
C-X-C Motif Chemokine Ligand 1	CXCL1	1,330	0,450	2,477	0,052
C-X-C Motif Chemokine Ligand 2	CXCL2	1,024	0,909	**1,351**	**0,032**
C-X-C Motif Chemokine Ligand 3	CXCL3	1,034	0,790	1,175	0,499
C-X-C Motif Chemokine Ligand 5	CXCL5	0,734	0,241	**0,638**	**0,017**
C-X-C Motif Chemokine Ligand 6	CXCL6	-	na	0,988	0,983
C-X-C Motif Chemokine Ligand 8	CXCL8	**3,566**	**0,013**	**4,451**	**0,012**
C-X-C Motif Chemokine Ligand 9	CXCL9	1,090	0,769	2,614	0,109
C-X-C Motif Chemokine Ligand 10	CXCL10	0,871	0,469	0,689	0,184
C-X-C motif chemokine receptor 1	CXCR1	0,833	0,216	0,605	0,521
C-X-C motif chemokine receptor 2	CXCR2	0,864	0,431	0,620	0,077
C-X-C motif chemokine receptor 4	CXCR4	-	na	-	na

Fas ligand	FASLG	0,686	0,629	0,438	0,507
Fos Proto-Oncogene	FOS	1,091	0,671	1,081	0,560
Interferon Gamma	IFNG	-	na	-	na
Interleukin 5	IL5	0,675	0,118	0,485	0,183
Interleukin 6	IL6	1,336	0,141	**1,708**	**0,011**
Interleukin 9	IL9	0,442	0,320	0,280	0,312
Interleukin 10	IL10	-	na	-	na
Interleukin 15	IL15	0,978	0,779	**0,768**	**0,016**
Interleukin 18	IL18	0,973	0,182	**0,891**	**0,020**
Interleukin 22	IL22	2,253	0,066	2,487	0,097
interleukin 10 receptor subunit beta	IL10RB	0,917	0,370	0,913	0,104
interleukin 17 receptor A	IL17A	-	na	-	na
interleukin 1 alpha	IL1A	0,834	0,356	1,049	0,525
interleukin 1 beta	IL1B	0,904	0,621	0,988	0,934
Interleukin 1 Receptor Type 1	IL1R1	1,003	0,983	1,032	0,792
Interleukin 1 Receptor Accessory Protein	IL1RAP	**0,816**	**0,008**	0,933	0,212
Interleukin 1 Receptor Antagonist	IL1RN	**1,062**	**0,047**	**0,683**	**0,002**
Interleukin 23 Subunit Alpha	IL23A	0,886	0,795	0,829	0,630
Interleukin 23 receptor	IL23R	1,752	0,212	1,003	0,998
Interleukin 6 Receptor	IL6R	1,022	0,912	0,955	0,431
Integrin Subunit Beta 2	ITGB2	0,912	0,554	0,963	0,716
Kininogen 1	KNG1	0,665	0,281	-	na
Lymphotoxin Alpha	LTA	0,372	0,384	0,409	0,466
Lymphotoxin Beta	LTB	1,537	0,293	1,495	0,258
Lymphocyte Antigen 96	LY96	1,165	0,456	**1,907**	**0,041**
MYD88 Innate Immune Signal Transduction Adaptor	MYD88	1,068	0,089	0,946	0,126
Nuclear Factor Kappa B Subunit 1	NFKB1	0,895	0,122	**0,833**	**0,040**
Nitric Oxide Synthase 2	NOS2	1,601	0,088	0,824	0,626
Nuclear Receptor Subfamily 3 Group C Member 1	NR3C1	0,947	0,432	**0,790**	**0,013**
Prostaglandin-Endoperoxide Synthase 2	PTGS2	1,015	0,786	0,979	0,609
Receptor Interacting Serine/Threonine Kinase 2	RIPK2	1,023	0,493	**1,145**	**0,008**
selectin E	SELE	0,455	0,123	0,396	0,120
TIR domain containing adaptor protein	TIRAP	1,081	0,638	1,144	0,243
toll like receptor 1	TLR1	0,781	0,345	**0,690**	**0,016**
toll like receptor 2	TLR2	0,983	0,837	1,167	0,054
toll like receptor 3	TLR3	0,946	0,498	**0,839**	**0,040**
toll like receptor 4	TLR4	1,192	0,756	0,729	0,789
toll like receptor 5	TLR5	0,824	0,062	0,987	0,885
toll like receptor 6	TLR6	**0,767**	**0,016**	1,046	0,533
toll like receptor 7	TLR7	1,172	0,515	1,455	0,123
toll like receptor 9	TLR9	2,267	na	0,790	na
tumor necrosis factor	TNF	0,809	0,763	0,576	0,568
TNF Superfamily Member 14	TNFSF14	**0,743**	**0,028**	0,484	0,085
Toll Interacting Protein	TOLLIP	0,899	0,444	0,886	0,087

Table S8. Mean fold changes in Inflammatory Response & Autoimmunity gene expression in HPrEC cells after treatment with 50 µg/ml of NaA nenozeolites modified with silane-PEG groups [NaA-silane-PEG] or NaA nanozeolites modified with silane-PEG groups and functionalized with anti-PSMA D2B antibodies [NaA-silane-PEG-D2B] for 24 hours. Mean fold change values from three independent experiments are presented. Fold changes statistically significant in Student's t-test are highlighted in green (up-regulated genes) or red (down-regulated genes). na – not analyzed, fold change and/or P-value cannot be computed due to very low expression level.

Target gene name	Gene	NaA-silane-PEG		NaA-silane-PEG-D2B	
		Mean fold change	P value	Mean fold change	p value
BCL6 transcription repressor	BCL6	1,190	0,101	**1,834**	**0,009**
complement C3	C3	1,108	0,618	1,112	0,386
complement C3a receptor 1	C3AR1	0,625	0,761	1,264	0,776
C-C motif chemokine ligand 2	CCL2	1,306	0,625	1,102	0,889
C-C motif chemokine ligand 3	CCL3	0,903	0,782	0,955	0,913
C-C motif chemokine ligand 4	CCL4	-	na	-	na
C-C motif chemokine ligand 5	CCL5	1,802	0,076	1,553	0,126
C-C motif chemokine ligand 7	CCL7	1,258	0,582	0,901	0,545
C-C motif chemokine ligand 8	CCL8	1,253	0,794	0,734	0,728
C-C motif chemokine ligand 11	CCL11	1,278	0,728	0,872	0,822
C-C motif chemokine ligand 13	CCL13	0,427	0,201	0,476	0,351
C-C motif chemokine ligand 16	CCL16	-	na	-	na
C-C motif chemokine ligand 17	CCL17	2,222	0,495	5,174	0,263
C-C motif chemokine ligand 19	CCL19	0,809	0,481	0,458	0,165
C-C motif chemokine ligand 21	CCL21	0,810	0,133	0,756	0,506
C-C motif chemokine ligand 22	CCL22	1,537	0,476	1,252	0,693
C-C motif chemokine ligand 23	CCL23	0,472	0,177	0,694	0,444
C-C motif chemokine ligand 24	CCL24	1,624	0,753	**6,188**	**0,030**
C-C Motif Chemokine Receptor 1	CCR1	1,317	0,598	1,773	0,478
C-C Motif Chemokine Receptor 2	CCR2	**0,195**	**0,017**	**3,900**	**0,015**
C-C Motif Chemokine Receptor 3	CCR3	0,920	0,843	1,218	0,774
C-C Motif Chemokine Receptor 4	CCR4	1,227	0,562	**0,453**	**0,035**
C-C Motif Chemokine Receptor 7	CCR7	0,268	0,104	**2,687**	**0,032**
NADH:ubiquinone oxidoreductase subunit A2	CD14	2,205	0,066	2,505	0,137
CD40 molecule	CD40	1,306	0,073	1,232	0,138
CD40 ligand	CD40LG	0,742	0,252	0,889	0,607
CCAAT Enhancer Binding Protein Beta	CEBPB	0,838	0,172	0,928	0,569
C-reactive protein	CRP	-	na	-	na
colony stimulating factor 1	CSF1	1,028	0,798	**1,324**	**0,033**
C-X-C Motif Chemokine Ligand 1	CXCL1	1,054	0,697	**0,393**	**0,000**
C-X-C Motif Chemokine Ligand 2	CXCL2	1,177	0,382	0,773	0,173
C-X-C Motif Chemokine Ligand 3	CXCL3	0,843	0,498	**1,375**	**0,011**
C-X-C Motif Chemokine Ligand 5	CXCL5	0,890	0,702	**0,528**	**0,034**
C-X-C Motif Chemokine Ligand 6	CXCL6	0,896	0,561	**0,243**	**0,004**
C-X-C Motif Chemokine Ligand 8	CXCL8	1,133	0,661	**0,777**	**0,022**
C-X-C Motif Chemokine Ligand 9	CXCL9	**0,527**	**0,005**	1,235	0,544
C-X-C Motif Chemokine Ligand 10	CXCL10	0,975	0,901	0,822	0,148
C-X-C motif chemokine receptor 1	CXCR1	0,995	0,989	1,357	0,412
C-X-C motif chemokine receptor 2	CXCR2	1,098	0,639	1,067	0,605
C-X-C motif chemokine receptor 4	CXCR4	1,190	na	1,116	na

Fas ligand	FASLG	0,858	0,785	1,162	0,832
Fos Proto-Oncogene	FOS	0,965	0,467	**0,751**	**0,026**
Interferon Gamma	IFNG	0,351	0,114	0,391	0,143
Interleukin 5	IL5	0,535	0,256	1,600	0,513
Interleukin 6	IL6	0,871	0,654	0,933	0,705
Interleukin 9	IL9	1,073	0,826	1,500	0,364
Interleukin 10	IL10	1,260	0,329	1,542	0,530
Interleukin 15	IL15	0,953	0,664	**2,801**	**0,000**
Interleukin 18	IL18	1,007	0,729	**1,282**	**0,015**
Interleukin 22	IL22	1,000	1,000	0,842	0,453
interleukin 10 receptor subunit beta	IL10RB	1,024	0,444	0,947	0,182
interleukin 17 receptor A	IL17A	1,113	0,891	0,970	0,974
interleukin 1 alpha	IL1A	0,833	0,072	0,876	0,054
interleukin 1 beta	IL1B	1,095	0,186	**0,678**	**0,007**
Interleukin 1 Receptor Type 1	IL1R1	**1,186**	**0,036**	1,035	0,270
Interleukin 1 Receptor Accessory Protein	IL1RAP	**0,858**	**0,027**	**0,652**	**0,000**
Interleukin 1 Receptor Antagonist	IL1RN	**1,446**	**0,000**	**0,496**	**0,000**
Interleukin 23 Subunit Alpha	IL23A	0,968	0,793	1,159	0,436
Interleukin 23 receptor	IL23R	0,998	0,996	2,306	0,131
Interleukin 6 Receptor	IL6R	1,172	0,308	**1,511**	**0,026**
Integrin Subunit Beta 2	ITGB2	1,675	0,585	0,390	0,413
Kininogen 1	KNG1	0,990	0,985	0,559	0,237
Lymphotoxin Alpha	LTA	0,673	0,324	0,978	0,949
Lymphotoxin Beta	LTB	0,674	0,212	0,575	0,202
Lymphocyte Antigen 96	LY96	**0,674**	**0,033**	**2,884**	**0,001**
MYD88 Innate Immune Signal Transduction Adaptor	MYD88	0,821	0,621	1,099	0,075
Nuclear Factor Kappa B Subunit 1	NFKB1	0,977	0,549	**0,692**	**0,033**
Nitric Oxide Synthase 2	NOS2	1,120	0,865	**2,603**	**0,029**
Nuclear Receptor Subfamily 3 Group C Member 1	NR3C1	1,041	0,263	**0,767**	**0,003**
Prostaglandin-Endoperoxide Synthase 2	PTGS2	0,919	0,523	**0,517**	**0,000**
Receptor Interacting Serine/Threonine Kinase 2	RIPK2	**0,900**	**0,020**	**0,798**	**0,006**
selectin E	SELE	0,634	0,065	0,646	0,261
TIR domain containing adaptor protein	TIRAP	0,756	0,063	0,820	0,266
toll like receptor 1	TLR1	0,875	0,658	0,656	0,089
toll like receptor 2	TLR2	1,307	0,050	**1,626**	**0,001**
toll like receptor 3	TLR3	0,984	0,866	**1,591**	**0,013**
toll like receptor 4	TLR4	1,229	0,670	1,529	0,434
toll like receptor 5	TLR5	1,510	0,064	0,971	0,848
toll like receptor 6	TLR6	**0,785**	**0,002**	**1,399**	**0,000**
toll like receptor 7	TLR7	1,046	0,946	0,824	0,662
toll like receptor 9	TLR9	2,246	0,377	1,774	0,545
tumor necrosis factor	TNF	1,718	0,096	1,698	0,104
TNF Superfamily Member 14	TNFSF14	0,833	0,366	0,790	0,413
Toll Interacting Protein	TOLLIP	1,036	0,453	0,824	0,057
